# Emergence of a Superplasmid Coharboring Hypervirulence and Multidrug Resistance Genes in Klebsiella pneumoniae Poses New Challenges to Public Health

**DOI:** 10.1128/spectrum.02634-22

**Published:** 2022-10-20

**Authors:** Xinmiao Jia, Ying Zhu, Peiyao Jia, Xiaoyu Liu, Wei Yu, Xue Li, Yingchun Xu, Qiwen Yang

**Affiliations:** a Department of Clinical Laboratory, State Key Laboratory of Complex Severe and Rare Diseases, Peking Union Medical College Hospital, Chinese Academy of Medical Sciences, Peking Union Medical College, Beijing, China; b Medical Research Center, State Key Laboratory of Complex Severe and Rare Diseases, Peking Union Medical College Hospital, Chinese Academy of Medical Sciences, Peking Union Medical College, Beijing, China; c Graduate School, Peking Union Medical College, Chinese Academy of Medical Sciences, Beijing, China; d Department of Clinical Laboratory, Beijing Anzhen Hospital, Capital Medical University, Beijing, China; Institut Pasteur

**Keywords:** carbapenemase, multidrug resistance, hypervirulence, *Klebsiella pneumoniae*, superplasmid

## Abstract

The emergence of plasmids coharboring hypervirulence (Hv) and multidrug resistance (MDR) genes has further accelerated the spread of MDR-Hv Klebsiella pneumoniae (MDR-HvKP) strains, having a severe impact on public health. Here, we report an MDR-Hv superplasmid coharboring hypervirulence and MDR genes and the detailed characterization of its genetic and phenotypic features. This plasmid was identified in an ST11 (sequence type 11)-K64 carbapenem-resistant hypervirulent K. pneumoniae (CR-HvKP) strain, SZS128, which was responsible for a bloodstream infection in a 21-year-old female. Susceptibility testing showed that SZS128 was resistant to amikacin, levofloxacin, and almost all of the β-lactams examined. SZS128 showed high virulence in a Galleria mellonella survival assay and a mouse intraperitoneal infection model. Genomic analysis showed that SZS128 not only possessed a KPC plasmid (pSZS128-KPC) but also carried a superplasmid (pSZS128-Hv-MDR) coharboring hypervirulence and MDR genes and possessing complete conjugative regions. Conjugation and transformation assays confirmed the potential for horizontal transfer and the high stability (retention rate of >95%) of the pSZS128-Hv-MDR superplasmid. Furthermore, growth curve assessment confirmed that there was no increase in the fitness cost in the presence of pSZS128-Hv-MDR. Therefore, we define a superplasmid as a plasmid fulfilling all the following criteria: (i) a single plasmid that coharbors hypervirulence and MDR genes, (ii) a plasmid that harbors complete conjugative elements that guarantee self-transmissibility, (iii) a plasmid that is stable and conserved, and (iv) a plasmid with no fitness cost to the host strain. The emergence of this kind of superplasmid could represent a serious threat to public health, and urgent control measures must be implemented.

**IMPORTANCE** This self-transmissible superplasmid, which simultaneously carries hypervirulence and MDR genes, greatly enhances the challenges to clinical prevention and control and anti-infection treatment. Thus, active surveillance of this type of superplasmid is needed to prevent these efficient resistance/virulence plasmids from disseminating in hospital settings. Our findings provide a reference for defining the term “superplasmid” and emphasize the importance of raising public awareness of the rapid dissemination of this self-transmissible superplasmid and the consistent emergence of MDR-HvKP strains.

## OBSERVATION

In recent years, the emergence of “superbugs,” which are resistant to most of the antibiotics and other medications commonly used to treat the infections that they cause, has become a public health concern worldwide (1, 2). Many different types of bacterial species have been described as superbugs, including carbapenem-resistant Klebsiella pneumoniae (CRKP) and extended-spectrum-β-lactamase (ESBL)-producing K. pneumoniae strains (3, 4). Moreover, some CRKP or ESBL-producing K. pneumoniae strains have also acquired various virulence factors, such as regulator of mucoid phenotype (*rmpA*) and siderophores (especially aerobactin), that confer hypervirulence (Hv) and have become hypervirulent K. pneumoniae (HvKP) strains (5–8). HvKP strains often cause life-threatening community-acquired infections in young and healthy hosts (5, 6). The emergence of carbapenem-resistant HvKP (CR-HvKP) and ESBL-producing HvKP strains is attributed mostly to the acquisition of hypervirulence and multidrug resistance (MDR) genes (6, 9, 10). These genes are usually harbored on different plasmids (11–13), but in recent years, plasmids containing both virulence genes and MDR genes have also been reported occasionally (14–16). The emergence of this kind of plasmid has further accelerated the simultaneous spread of hypervirulence genes and MDR genes, having a more serious impact on public health.

In this study, we identified an ST11-K64 clinical CR-HvKP strain, SZS128, carrying a “superplasmid,” and we report its detailed structural characterization here. We define plasmids with the following characteristics as superplasmids: (i) a single plasmid that coharbors hypervirulence genes and MDR genes, (ii) a plasmid that harbors complete conjugative elements that guarantee self-transmissibility, (iii) a plasmid that is stable and conserved, and (iv) a plasmid with no fitness cost to the host strain.

The clinical K. pneumoniae strain SZS128 was isolated from a blood culture collected from a 21-year-old female in Shanghai, China, on 8 May 2018. The patient was hospitalized with a lower respiratory infection and had a fever of >39°C that lasted for more than 14 days. The total white blood cell (WBC) count, neutrophil (NEU) percentage, C-reactive protein (CRP) level, and procalcitonin (PCT) level were 4.75 × 10^9^ cells/L, 92.9%, 7.8 mg/L, and 0.9 ng/mL, respectively. During hospitalization, the patient developed a bloodstream infection and acute liver failure and was admitted to the intensive care unit (ICU) and treated with meropenem (1 g every 8 h [q8h] for 4 days), vancomycin (1 g q12h for 1 day), and polymyxin B (1,500,000 U daily for 3 days). Surgical intervention was performed during the treatment, but unfortunately, the patient had not healed upon discharge.

**FIG 1:**
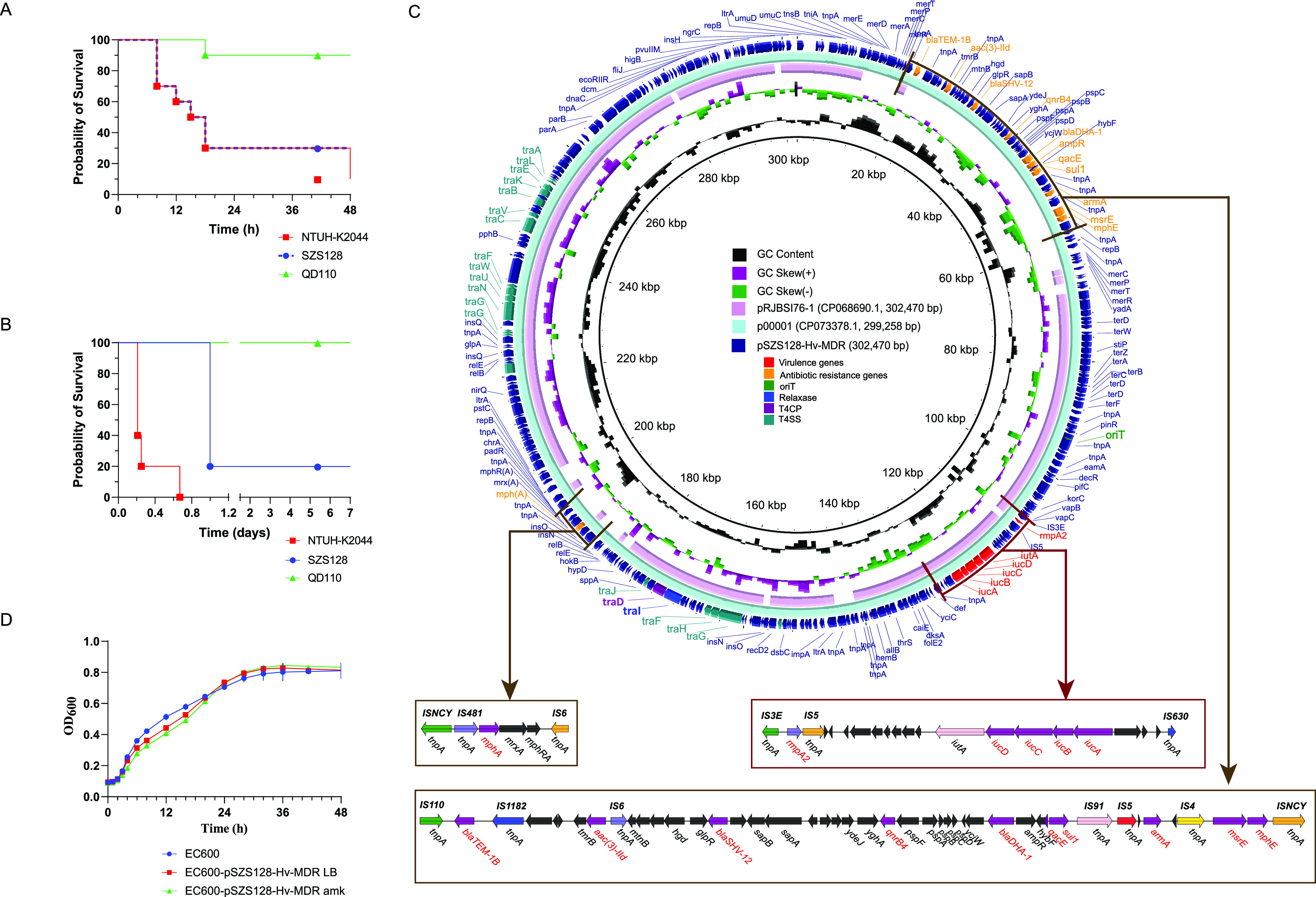
Virulence phenotype of CR-HvKP strain SZS128 and features of the superplasmid pSZS1280-Hv-MDR. (A and B) Virulence phenotype of SZS128 in the G. mellonella survival assay (A) and the mouse i.p. infection model (B). NTUH-K2044 was used as an Hv control strain, and QD110 was used as a low-virulence control strain. (C) Structural comparison of the pSZS1280-Hv-MDR superplasmid. The structural comparison was conducted among pSZS1280-Hv-MDR, p00001 (GenBank accession number CP073378.1), and pRJBSI76-1 (accession number CP068690.1). Alignments of similar plasmids are shown as concentric rings. The outermost ring shows the main coding genes of pSZS1280-Hv-MDR. The MDR region and hypervirulence region were further analyzed in detail. Antimicrobial resistance genes are highlighted in orange. Hypervirulence genes are highlighted in red. Conjugative elements are also highlighted, with *oriT* in green, the relaxase in blue, the T4CP in purple, and the T4SS in turquoise. (D) Growth curve comparison. Growth curves of the recipient strain E. coli EC600 and the transconjugants harboring the superplasmid pSZS1280-Hv-MDR with and without amikacin (*P* > 0.05) are shown. EC600 indicates the recipient strain E. coli EC600. EC600-pSZS1280-Hv-MDR LB indicates the transconjugant harboring plasmid pSZS1280-Hv-MDR in antibiotic-free LB broth. EC600-pSZS1280-Hv-MDR amk indicates the transconjugant harboring the plasmid pSZS1280-Hv-MDR on LB agar plates containing amikacin. OD_600_, optical density at 600 nm.

The results of susceptibility testing showed that isolate SZS128 was resistant to amikacin, levofloxacin, and almost all of the β-lactams examined, including cephalosporins (cefoxitin, ceftriaxone, ceftazidime, and cefepime), carbapenems (imipenem, meropenem, and ertapenem), combinations of β-lactams and β-lactamase inhibitors (piperacillin-tazobactam and ceftolozane-tazobactam), and aztreonam (see Table S1 in the supplemental material). The strain was susceptible to only imipenem-relebactam, ceftazidime-avibactam, and colistin (Table S1). To test the virulence of SZS128, the Galleria mellonella survival assay was performed and a mouse intraperitoneal (i.p.) infection model was established to compare the survival rates among NTUH-K2044 (an Hv control strain) (17), QD110 (a low-virulence control strain) (7), and SZS128. SZS128 showed high virulence in the G. mellonella survival assay and the mouse i.p. infection model (Fig. 1A and B).

To reveal the genetic basis of the MDR and hypervirulence phenotypes, we obtained the complete genome sequence of SZS128, including a 5.4-Mb chromosome, a 302-kb plasmid (pSZS128-Hv-MDR), a 51-kb plasmid (pSZS128-KPC), and a 10-kb plasmid (pSZS128-ccl) (Table 1). Bioinformatics analysis provided general information on the chromosome sequence, including the GC content (57.40%), the number of predicted protein-coding genes (5,291), the average gene length (902 bp), and the coding region proportion (88.87%). The three plasmids had shorter average gene lengths, lower coding region proportions, and lower GC contents. Sequence type (ST) and serotype analysis showed that SZS128 belongs to ST11 and K64.

**TABLE 1:** Genomic characteristics and antimicrobial resistance/virulence genotype analysis of the chromosome and plasmid sequences

Sequence name^*a*^	Genome size (bp)	GC content (%)	No. of coding genes	Avg gene size (bp)	Coding region size (bp) (% of sequence)	No. of tRNAs	No. of rRNAs	Virulence genes	Drug resistance genes
SZS128_chr	5,369,699	57.40	5,291	902	4,771,827 (88.87)	85	25	*iutA*, *mrkABCDFHIJ*, *fyuA*, *irp*, *ybtAEPQSTUX*	*bla*_SHV-182_, *fosA*
pSZS128-Hv-MDR	302,470	47.89	330	751	247,668 (81.88)			*rmpA2*, *iucABCD*, *iutA*	*qacE*, *qnrB4*, *armA*, *aac(3)-IId*, *bla*_SHV-12_, *bla*_TEM-1B_, *bla*_DHA-1_, *sul1*, *mphA*, *mphE*, *msrE*
pSZS128-KPC	51,876	54.09	68	596	40,503 (78.08)				*bla*_KPC-2_, *bla*_CTX-M-65_, *catA2*
pSZS128-ccl	10,060	55.06	13	577	7,500 (74.55)				

a Chromosomal sequence names end with the term “chr”; plasmid sequence names begin with the letter “p.”

Virulence gene (Table 1) analysis showed that SZS128 contains 25 virulence genes, with 19 located on the 5.2-Mb chromosome, including the genes encoding the ferric aerobactin receptor protein IutA and the type III fimbria proteins MrkABCDFHIJ and the yersiniabactin-related genes *ybtAEPQSTUX*, *irp*, and *fyuA*, and 6 located on the 302-kb plasmid pSZS128-Hv-MDR, including 5 hypervirulence marker genes (the regulator of mucoid phenotype gene *rmpA2* and the aerobactin-related genes *iucABCD*) (11) and *iutA*. No virulence genes were discovered on the other two plasmids, namely, pSZS128-KPC and pSZS128-ccl.

Sixteen antimicrobial resistance genes were found in SZS128 (Table 1), including 2 antimicrobial resistance genes on the chromosome (*fosA*, a glutathione transferase-encoding gene, and *bla*_SHV-182_, a β-lactamase-encoding gene), 11 antimicrobial resistance genes on the 302-kb plasmid pSZS128-Hv-MDR {3 β-lactamase genes (*bla*_SHV-12_, *bla*_TEM-1B_, and *bla*_DHA-1_), 2 aminoglycoside resistance genes [*armA* and *aph(3″)-Ib*], 1 sulfonamide resistance gene (*sul1*), 1 quinolone resistance gene (*qnrB4*), 1 disinfectant resistance gene (*qacE*), and 3 macrolide resistance genes [*mphA*, *mphE*, and *msrE* (among these, *msrE* is also a lincosamide and streptogramin B resistance gene)]}, and 3 antimicrobial resistance genes on the 51-kb plasmid pSZS128-KPC (1 carbapenemase gene [*bla*_KPC-2_], 1 β-lactamase gene [*bla*_CTX-M-65_], and 1 phenicol resistance gene [*catA2*]). No antimicrobial resistance genes were found on the 10-kb plasmid (pSZS128-ccl).

Through the above-described analysis, we found that the pSZS128-Hv-MDR plasmid contained not only hypervirulence genes but also MDR genes. The complete nucleotide sequence of the circular pSZS128-Hv-MDR plasmid was 302,470 bp in length, with an average GC content of 47.89% and with 330 open reading frames (Table 1). This plasmid contained two types of plasmid replication initiation genes: IncFIB and IncHI1B. Further structural analysis showed that the drug resistance region of the plasmid can be divided into two parts: part I contained 10 [*qacE*, *qnrB4*, *armA*, *aac(3)-IId*, *bla*_SHV-12_, *bla*_TEM-1B_, *bla*_DHA-1_, *sul1*, *mphE*, and *msrE*] of the 11 antimicrobial resistance genes and was concentrated in an ~40-kb region from bp 18658 to bp 57843, and part II contained 1 macrolide resistance gene, *mphA*, which was located in the region from bp 189071 to bp 195649 (Fig. 1C). Further mobile element analysis showed that part I was arranged as IS*110–bla*_TEM-1B_*–*IS*1182–aac(3)-IId–*IS*6–bla*_SHV-12_*–qnrB4–bla*_DHA-1_*–qacE–sul1–*IS*91–*IS*5–armA–*IS*4–msrE–mphE–*IS*NCY* and that part II was arranged as IS*NCY*-IS*481*-*mphA*-IS*6* (Fig. 1C). The existence of these antimicrobial resistance genes severely limits the availability of effective antibiotics and poses a serious hazard to human health. Virulence genes (*rmpA2*, *iucABCD*, and *iutA*) were located in the region from bp 108031 bp to bp 126778, arranged as IS*3E*-*rmpA2*-IS*5*-*iutA*-*iucD*-*iucC*-*iucB*-*iucA*-IS*630* (Fig. 1C).

We also analyzed the conjugative regions of pSZS128-Hv-MDR and found that it contained four complete modules of self-transmissible plasmids (18), namely, the origin-of-transfer site (*oriT*), a relaxase gene (*traI*), a gene encoding the type IV coupling protein (T4CP) (*traD*), and a gene cluster for the bacterial type IV secretion system (T4SS) (*traABCEFGHJKLNUVW*) (Fig. 1C), indicating its self-transmissibility. Additionally, a full-plasmid comparative analysis of pSZS128-Hv-MDR was conducted using NCBI BLAST, and it exhibited 99% identity with plasmid p00001 (GenBank accession number CP073378.1) from Klebsiella sp. strain P1927 (Fig. 1C and Fig. S1), suggesting that this superplasmid is highly conserved.

To evaluate the transferability of the pSZS128-Hv-MDR superplasmid, conjugation assays were performed by coculturing SZS128 with Escherichia coli EC600. The results indicated that the pSZS128-Hv-MDR plasmid harboring hypervirulence genes and MDR genes in the clinical strain SZS128 was self-transmissible, which is consistent with the results of the conjugative region analysis. Moreover, the hypervirulence genes and MDR genes were also transferred along with the plasmid, which was confirmed by PCR. As shown by the susceptibility results in Table S1, the transconjugant acquired resistance via the plasmid pSZS128-Hv-MDR, exhibiting resistance to amikacin, aztreonam, cefoxitin, ceftriaxone, and ceftazidime.

The stability of the pSZS128-Hv-MDR plasmid was also evaluated during serial passage in the laboratory for 15 days. It displayed high stability, as the retention rates were still over 95% at the end of the experiment, which was confirmed by PCR and culture on selection plates containing drugs. The colonies on single-antibiotic plates were similar to those on double-antibiotic plates. Moreover, the PCR assay verified that the drug resistance of the strain was caused by the corresponding gene, and all of the colonies contained two drug resistance genes, indicating that the plasmid was very stable.

Furthermore, the biological fitness cost of acquiring plasmids harboring hypervirulence genes and MDR genes was evaluated. Interestingly, no significant differences in growth rates were observed between the recipient strain EC600 and the transconjugant harboring pSZS128-Hv-MDR cultured in Luria-Bertani (LB) broth with or without amikacin (*P* > 0.05) (Fig. 1D). Therefore, the hypervirulence and antibiotic resistance conferred by pSZS128-Hv-MDR did not increase the growth fitness cost for the resistant strains compared to their susceptible counterparts.

In addition to the plasmid pSZS128-Hv-MDR coharboring hypervirulence and MDR genes, the carbapenemase resistance plasmid pSZS128-KPC was also present in strain SZS128. Detailed information can be found in Fig. S2 and S3 in the supplemental material.

In this study, we identified an ST11-K64 clinical CR-HvKP strain, SZS128, that causes bloodstream infection. SZS128 not only possessed a KPC plasmid but also carried a superplasmid coharboring hypervirulence genes and ESBL genes and possessing complete conjugative regions (Table S2). This superplasmid was self-transmissible and stable. Moreover, it did not increase the growth fitness cost for the isolate SZS128. This self-transmissible superplasmid greatly increases the challenges in clinical prevention and control and anti-infection treatment. Thus, active surveillance of this type of superplasmid is needed to prevent such efficient resistance/virulence plasmids from disseminating in hospital settings. Our findings provide a reference for defining the term “superplasmid” and emphasize the importance of raising public awareness of the rapid dissemination of this self-transmissible superplasmid and the consistent emergence of CR-HvKP strains.

The study protocol was reviewed by the human research ethics committee of the Institutional Review Board (IRB) of the Peking Union Medical College Hospital. This project did not affect the normal diagnosis and treatment of patients; after consultation with the IRB, formal ethical approval was reviewed and waived, and written patient consent was not required (ethics approval number S-K467). The animal protocols were reviewed and approved by the Animal Ethics Committee and Administration Institutional Animal Care Committee of Tsinghua University. All experiments were carried out in the Tsinghua University animal biosafety level 2 (ABSL-2) facility under the guidelines of the ethics of animal experimentation statement.

### Data availability.

The complete genome sequences, including the chromosome and three plasmids, of strain SZS128 were deposited in GenBank with accession numbers CP099522 to CP099525.
