# Supplementary material for: Emergence of a Superplasmid Coharboring Hypervirulence and Multidrug Resistance Genes in Klebsiella pneumoniae Poses New Challenges to Public Health
Source: Microbiol Spectr. 2022 Oct 20;10(6):e02634-22. doi: 10.1128/spectrum.02634-22 (PMC9769819; doi:10.1128/spectrum.02634-22)
Supplement: Supplemental file 1 — Supplemental methods, Tables S1 and S2, and Fig. S1 to S3. Download spectrum.02634-22-s0001.pdf, PDF file, 1.0 MB [file spectrum.02634-22-s0001.pdf]

SUPPLEMENTARY MATERIALS

Characterization of the carbapenemase resistance plasmid pSZS128-KPC. Except for the plasmid pSZS128-Hv-MDR coharboring hypervirulence genes and multidrug resistance genes, the carbapenemase resistance plasmid pSZS128-KPC was also present in strain SZS128. This plasmid (pSZS128-KPC) is a IncFII-type plasmid that is 51,876 bp in length and circular, with an average GC content of 54.09% and 68 open reading frames (Table 1). Full-plasmid BLAST analysis showed that pSZS128-KPC shared 100% identity with pKPC2_020002 (CP028541.2) and pBSI014-KPC2 (MT2698221.1), and these plasmids showed 100% coverage (Figure S2). Further plasmid structure analysis showed that pSZS128-KPC harbored only the main drug resistance region of pKPC2_020002 or pBSI014-KPC2 and lost many plasmid maintenance- and conjugal transfer-related genes (Figure S3). There was only one copy of *bla*$_{KPC-2}$ in pSZS128-KPC, while there were three copies in pKPC2_020002 and two copies in pBSI014-KPC2.

SUPPLEMENTARY MATERIALS AND METHODS

Bacterial strains. The strain was isolated on May 8, 2018, from a blood specimen obtained from a 21-year-old female who presented with acute hepatic failure in Shanghai, China, and was identified by the Vitek MS MALDI-TOF (BioMérieux) system at Peking Union Medical College Hospital. The hemogram indexes are the results of the first examination after admission within 1-3 days of the occurrence of a bloodstream infection. The rifampicin-resistant *E. coli* strain EC600 was used in the transconjugation experiments.

Antimicrobial susceptibility testing. We used the broth microdilution method for antimicrobial susceptibility testing as per the Clinical and Laboratory Standards Institute (CLSI) recommendations (1). Minimum inhibitory concentrations (MICs) were interpreted according to the CLSI M100-S30 guidelines (1). *E. coli* ATCC 25922, *Pseudomonas aeruginosa* ATCC 27853, and *K. pneumoniae* ATCC700603 were used as quality controls.

Virulence phenotypic detection. To test the virulence of SZS128, larval and mouse intraperitoneal (IP) infection models were established by comparing the survival rates among the HvKP reference strain NTUH-K2044 (a hypervirulent control strain) (2), the classic *K. pneumoniae* strain QD110 (a low-virulence control strain) (3) and SZS128. QD110 contained no virulence genes in the virulence factor database (VFDB) based on the assembled contigs.

For the *G. mellonella* survival assay (4), 5–6 standardized instar larvae that were 2–3 cm in length with good activity and a creamy color were picked to perform this test. The larvae of *G. mellonella* were inoculated with 10 μL of the *K. pneumoniae* isolate SZS128, 10 μL of the HvKP reference strain NTUH-K2044, or 10 μL of the classic *K. pneumoniae* strain QD110 at a cell density of 1×10^7 CFU/mL via injection into the last

left proleg. The larvae were observed for survival at 12 h, 15 h, 18 h, 24 h, 36 h, and 48 h after bacterial inoculation. Each treatment group had 10 larvae.

Mouse IP infection models were established as described in previously published studies with modifications (5, 6). Bacteria were grown in LB broth until the logarithmic phase and stored at -80°C after adding an equal volume of 50% glycerol. Then, the bacterial cells were washed with PBS. Five ICR female mice at the age of six or seven weeks (Vital River) were injected with approximately $5\sim8\times10^7$ CFU of NTUH-K2044, QD110, or SZS128. Then, the mortality of the mice was observed for up to seven days.

Survival rates were calculated and visually represented using GraphPad Prism 7 (GraphPad, La Jolla, USA). The log-rank (Mantel–Cox) test was used to compare survival rates among NTUH-K2044, QD110, and SZS128. P values <0.05 were considered statistically significant.

Genomic DNA extraction, sequencing, assembly, correction, and annotation. An UltraClean® Microbial DNA Isolation Kit (MOBIO Laboratories, Inc.) was used for genomic DNA extraction. Whole-genome sequencing was implemented using the PacBio Sequel platform. A 10 kb SMRTbell library was prepared from sheared genomic DNA (≥5 g) with an additional bead clean-up step before primer annealing.

To correct the polymerization errors generated during PacBio sequencing, we also resequenced this isolate using the Illumina sequencing platform. Paired-end libraries were constructed from 5 μg of isolated genomic DNA using a TruSeq DNA Sample Prep Kit (Illumina Inc., San Diego, California, USA) and sequenced using the Illumina platform with a read length of 2×150 bp. A threshold of 0.01 (Phred score of 20) was used to filter raw reads. Genome assembly from short and long sequencing reads was conducted using Unicycler (7).

Genome sequences were initially annotated with the rapid prokaryotic genome

annotation software Prokka (8) and further annotated by BLAST searches against the RefSeq and UniProtKB/Swiss-Prot databases. Pairwise sequence comparisons were also performed using BLAST. All mobile elements were identified using ExPASy (9), ISFinder (10), the Transposon Registry (11), INTEGRALL (12), and Integron Finder (https://github.com/gem-pasteur/Integron_Finder). Comparisons of plasmid structures were conducted to analyze the sequence homology. BRIG software was used for the generation of circular plasmid structure maps.

Sequence type (ST), serotype, virulence genes, antimicrobial resistance genes, and conjugative region analysis. STs were determined using SRST2 (13) based on the Illumina reads. The serotype was analyzed using Kaptive (14) based on the assembled genome. Virulence gene sequences were downloaded from the Virulence Factor Database (VFDB). FASTA sequences of the downloaded virulence genes were used for searching for corresponding genes by BLAST with coverage of 50% and identity of 90%. Antimicrobial resistance genes were analyzed through ResFinder 4.0 based on the whole-genomic sequences. The conjugative regions of self-transmissible plasmids typically consist of four modules: the origin of transfer site (*oriT*), relaxase gene, gene encoding the type IV coupling protein (T4CP), and gene cluster for the bacterial type IV secretion system (T4SS); these were predicted using oriTfinder (15).

Plasmid conjugation assay. The transferability of pSZS128-Hv-MDR among isolates was determined using SZS128 as the donor and *E. coli* EC600 as the recipient in a conjugation assay. The conjugation procedure was performed according to a previously described protocol with slight modification (16). One hundred microliters of overnight culture of the clinical isolate (SZS128) in LB broth containing amikacin (50 μg/ml) and 300 μl of overnight culture of the recipient EC600 in LB broth containing rifampicin (100 μg/ml) were mixed, washed three times with PBS, added to

5 ml of antibiotic-free brain-heart infusion (BHI) broth and incubated at 37°C for 18 h. Then, the conjugant mixtures were placed on rifampin (300 μg/ml)-containing BHI agar or rifampin (300 μg/ml)- and amikacin (50 μg /ml)-containing BHI agar. Transfer frequencies were calculated by dividing the number of colonies on rifampin- and amikacin-containing BHI agar by the number of colonies on rifampin-containing BHI agar. PCR analysis and agarose gel electrophoresis were performed to confirm the presence of the plasmid by detecting the bla_{TEM} gene (primer sequences (5′-3′): forward, GAGTGGGTTACATCGAACTGG; reverse, TCCATAGTTGCCTGACTCCC) in the conjugants.

Plasmid stability testing. The stability of the two plasmids in SZS128 was assessed through sequential passages in antibiotic-free LB broth at 37°C and 180 rpm once per day for 15 days at a 1:100 dilution (17). On the 15th day, the culture was serially diluted and plated onto LB plates with meropenem (4 mg/L) to select for pSZS128-KPC and/or amikacin (50 mg/L) to select for pSZS128-Hv-MDR. Colonies that grew on the plates were also further analyzed for the presence of bla_{KPC} and $bla_{\mathrm{TEM\text{-}1B}}$ by PCR. Sixteen colonies from each plate were randomly selected and examined by PCR (bla_{KPC} primer sequences (5′-3′): forward TGTCACTGTATCGCCGTC, reverse CTCAGTGCTCTACAGAAAACC; bla_{TEM} primer sequenced (5′-3′): forward GAGTGGGTTACATCGAACTGG, reverse TCCATAGTTGCCTGACTCCC) and agarose gel electrophoresis.

Fitness cost assessment. A growth curve was used to assess the fitness impact of plasmid carriage under noncompetitive conditions as described previously by Wang et al (18). The recipients and transconjugants carrying the pSZS128-Hv-MDR plasmid from the SZS128 isolate were cultured overnight in LB broth without or with amikacin at 37°C. Bacterial suspensions were diluted 1:1000 in LB medium (approximately 10^6

CFU/ml) and grown at 37°C in triplicate for 36 h in an Epoch™ 2 Microplate Spectrophotometer from BioTek Instruments. The OD600 of each culture was measured every 30 min, and the plates were shaken for 15 s before measurement. GraphPad Prism version 8 (GraphPad Software, Inc., USA) was used to estimate the growth curves. Statistical significance was determined for overall error at the 0.05 level (95% confidence interval) using the Friedman test with Dunn's multiple comparisons test.

SUPPLEMENTARY TABLES

Table S1 MIC of the clinical strain SZS128 and EC600 with/without plasmid pSZS128-Hv-MDR.

Antimicrobial agents (MIC µg/ml)	SZS128	EC600	EC600-pSZS128-Hv-MDR
Colistin	≤0.5	≤0.5	≤0.5
Amikacin	>32	≤ 4	>32
Aztreonam	>16	≤ 0.5	>16
Cefoxitin	>16	≤2	>16
Ceftriaxone	>8	≤0.5	8
Ceftazidime	>16	≤ 0.5	>16
Cefepime	>16	≤0.5	2
Levofloxacin	>4	≤0.25	2
Meropenem	>8	≤0.06	0.12
Imipenem	>8	0.25	0.5
Ertapenem	>4	≤0.06	0.12
Ceftazidime-avibactam	1/4	0.25/4	0.5/4
Piperacillin-tazobactam	>64/4	≤2/4	≤4/4
Imipenem-relebactam	1/4	0.25/4	0.25/4
Ceftolozane-tazobactam	>8/4	0.5/4	2/4

Table S2 Characteristics of the super-plasmid pSZS128-Hv-MDR.

Characters	Phenotype and Genotype	
Hypervirulence	high virulence in *G. mellonella* survival assay and mouse intraperitoneal infection model	*rmpA2* and *iucABCD*
MDR	β-lactamase, aminoglycoside, sulfonamide, quinolone, disinfectant, macrolide, lincosamide and streptogramin B resistance	*bla*$_{SHV-12}$, *bla*$_{TEM-1B}$, *bla*$_{DHA-1}$, *armA*, *aph(3")-Ib*, *sul1*, *qnrB4*, *qacE*, *mphA*, *mphE*, and *msrE*
Conjugative elements	*oriT*, relaxase gene (*traI*), T4CP (*traD*), T4SS (*traABCEFGHJKLNUVW*)	
Self-transmissible ability	Yes	
Plasmid stability and conservation	High stability and conservation	
Fitness cost	No	

SUPPLEMENTARY FIGURES

Figure S1

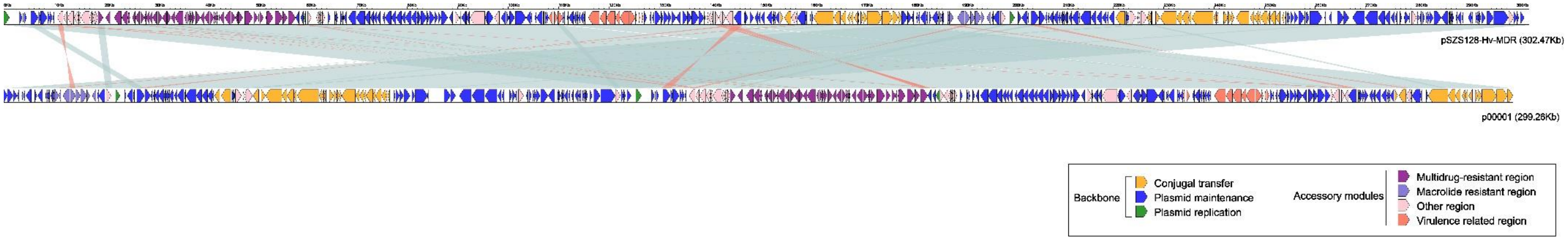

Figure S1. Linear comparison of plasmid pSZS1280-Hv-MDR with p00001 (CP073378.1) and pRJBSI76-1 (CP068690.1). Genes are denoted by arrows. Genes, mobile elements, and other features are colored based on functional classification. Shading denotes the regions with high homology (95% nucleotide identity).

Figure S2

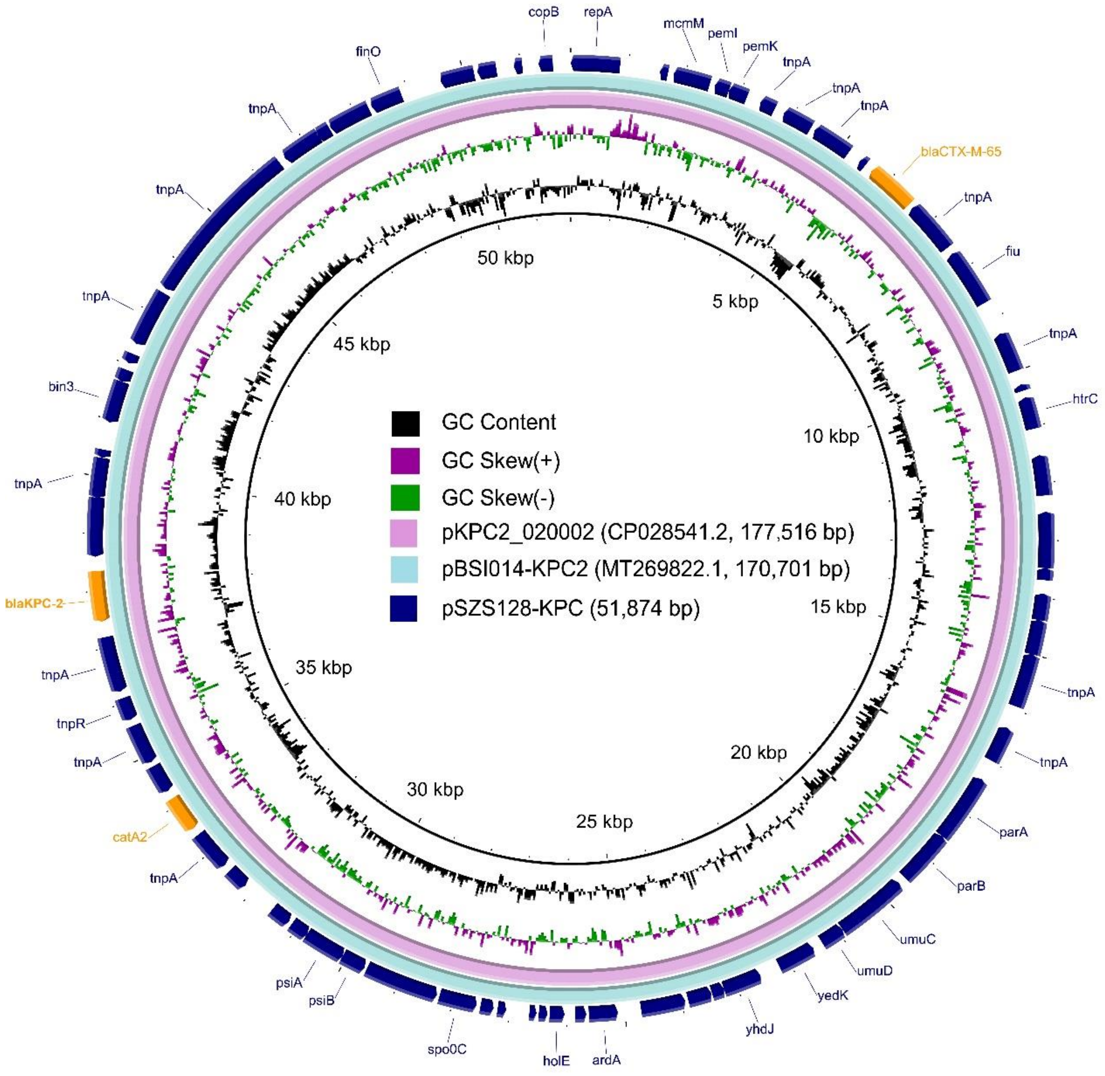

Figure S2. Structural comparison of pSZS128-KPC with pKPC2_020002 and pBSI014-KPC2. Alignments of resemble plasmids are shown as concentric rings. The outermost shows the main coding genes of pSZS128-KPC. *Bla*$_{\text{KPC-2}}$, *bla*$_{\text{CTX-M-65}}$ and *catA2* are highlighted in orange.

Figure S3

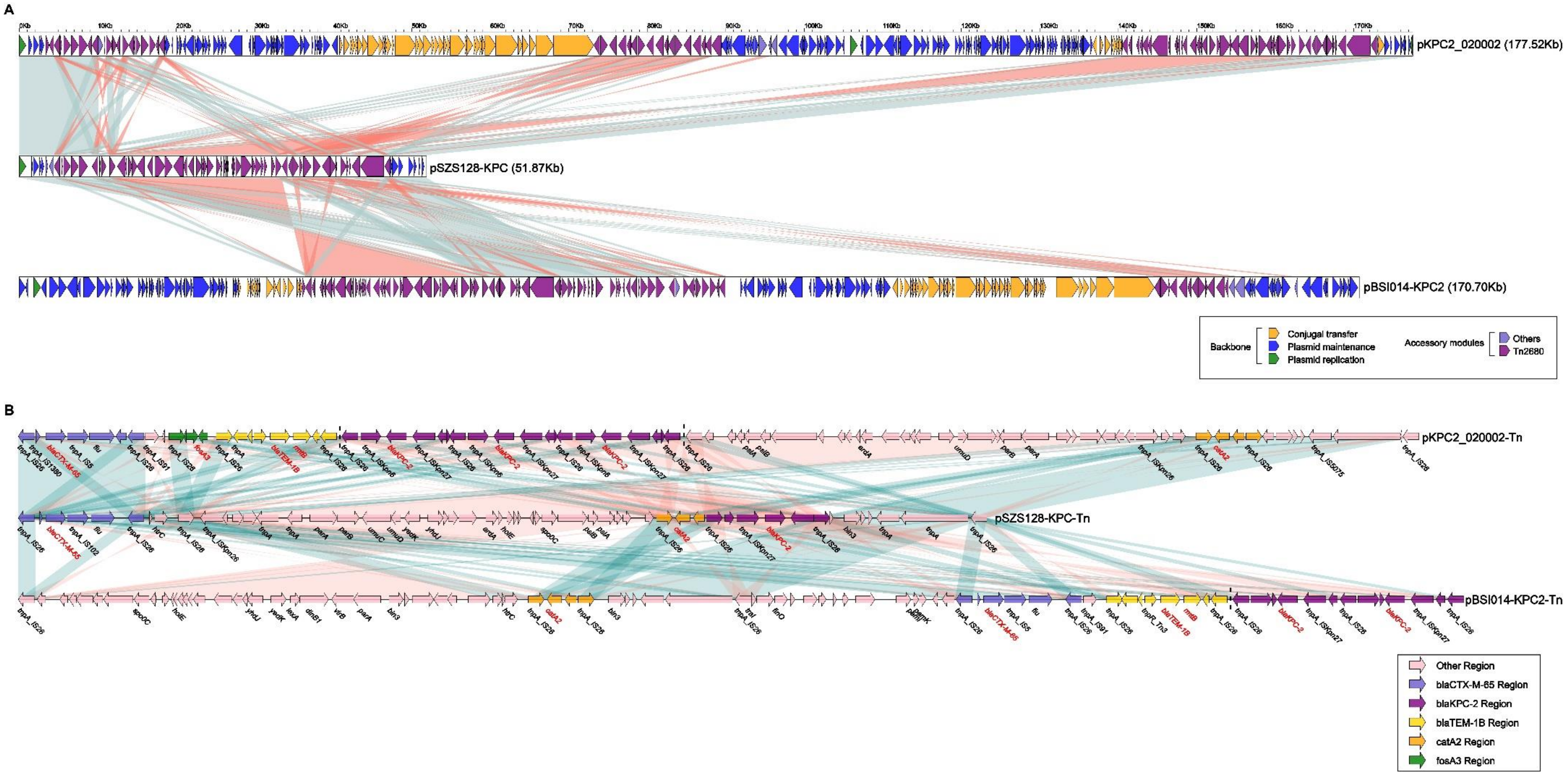

A
pKPC2_020002 (177.52Kb)
pSZS128-KPC (51.87Kb)
pBSI014-KPC2 (170.70Kb)
Backbone
Conjugal transfer
Plasmid maintenance
Plasmid replication
Accessory modules
Others
Tn2680
B
pKPC2_020002-Tn
pSZS128-KPC-Tn
pBSI014-KPC2-Tn
Other Region
blaCTX-M-65 Region
blaKPC-2 Region
blaTEM-1B Region
catA2 Region
fosA3 Region

Figure S3. Structure of plasmid pSZS128-KPC and antimicrobial-resistance genes related regions. (A) Linear comparison of plasmid pSZS128-KPC with two similar plasmids (pKPC2_020002, and pBSI014-KPC2). (B) Linear genomic comparison of the drug resistance region in plasmids among pSZS128-KPC, pKPC2_020002, and pBSI014-KPC2. Antimicrobial-resistance genes are highlighted in red. Genes are denoted by arrows. Genes, mobile elements, and other features are colored based on functional classification. Shading denotes the regions with high homology (95% nucleotide identity).

REFERENCES

1. CLSI. 2020. Performance standards for antimicrobial susceptibility testing; twenty-eight informational supplement. CLSI document M100-S30 Clinical and Laboratory Standards Institute: Wayne, PA.
2. Wu KM, Li LH, Yan JJ, Tsao N, Liao TL, Tsai HC, Fung CP, Chen HJ, Liu YM, Wang JT, Fang CT, Chang SC, Shu HY, Liu TT, Chen YT, Shiau YR, Lauderdale TL, Su IJ, Kirby R, Tsai SF. 2009. Genome sequencing and comparative analysis of *Klebsiella pneumoniae* NTUH-K2044, a strain causing liver abscess and meningitis. J Bacteriol 191:4492-4501.
3. Yang Q, Jia X, Zhou M, Zhang H, Yang W, Kudinha T, Xu Y. 2020. Emergence of ST11-K47 and ST11-K64 hypervirulent carbapenem-resistant *Klebsiella pneumoniae* in bacterial liver abscesses from China: a molecular, biological, and epidemiological study. Emerg Microbes Infect 9:320-331.
4. Liu E, Jia P, Li X, Zhou M, Kudinha T, Wu C, Xu Y, Yang Q. 2021. In vitro and in vivo Effect of Antimicrobial Agent Combinations Against Carbapenem-Resistant *Klebsiella pneumoniae* with Different Resistance Mechanisms in China. Infect Drug Resist 14:917-928.
5. Russo TA, MacDonald U, Hassan S, Camanzo E, LeBreton F, Corey B, McGann P. 2021. An Assessment of Siderophore Production, Mucoviscosity, and Mouse Infection Models for Defining the Virulence Spectrum of Hypervirulent *Klebsiella pneumoniae*. mSphere 6:e00045-21.
6. Tian D, Wang W, Li M, Chen W, Zhou Y, Huang Y, Sheng Z, Jiang X. 2021.

Acquisition of the Conjugative Virulence Plasmid From a CG23 Hypervirulent *Klebsiella pneumoniae* Strain Enhances Bacterial Virulence. Front Cell Infect Microbiol 11:752011.

7. Wick RR, Judd LM, Gorrie CL, Holt KE. 2017. Unicycler: Resolving bacterial genome assemblies from short and long sequencing reads. PLoS Comput Biol 13:e1005595.

8. Seemann T. 2014. Prokka: rapid prokaryotic genome annotation. Bioinformatics 30:2068-2069.

9. Artimo P, Jonnalagedda M, Arnold K, Baratin D, Csardi G, de Castro E, Duvaud S, Flegel V, Fortier A, Gasteiger E, Grosdidier A, Hernandez C, Ioannidis V, Kuznetsov D, Liechti R, Moretti S, Mostaguir K, Redaschi N, Rossier G, Xenarios I, Stockinger H. 2012. ExPASy: SIB bioinformatics resource portal. Nucleic Acids Res 40:W597-W603.

10. Siguier P, Perochon J, Lestrade L, Mahillon J, Chandler M. 2006. ISfinder: the reference centre for bacterial insertion sequences. Nucleic Acids Res 34:D32-D36.

11. Tansirichaiya S, Rahman MA, Roberts AP. 2019. The Transposon Registry. Mob DNA 10:40.

12. Moura A, Soares M, Pereira C, Leitao N, Henriques I, Correia A. 2009. INTEGRALL: a database and search engine for integrons, integrases and gene cassettes. Bioinformatics 25:1096-1098.

13. Inouye M, Dashnow H, Raven LA, Schultz MB, Pope BJ, Tomita T, Zobel J,

Holt KE. 2014. SRST2: Rapid genomic surveillance for public health and hospital microbiology labs. Genome Med 6:90.

14. Wyres KL, Wick RR, Gorrie C, Jenney A, Follador R, Thomson NR, Holt KE. 2016. Identification of Klebsiella capsule synthesis loci from whole genome data. Microb Genom 2:e000102.

15. Li X, Xie Y, Liu M, Tai C, Sun J, Deng Z, Ou HY. 2018. oriTfinder: a web-based tool for the identification of origin of transfers in DNA sequences of bacterial mobile genetic elements. Nucleic Acids Res 46:W229-W234.

16. Jia X, Jia P, Zhu Y, Yu W, Li X, Xi J, Liu X, Liao K, Xu Y, Cheng B, Yang Q. 2022. Coexistence of blaNDM-1 and blaIMP-4 in One Novel Hybrid Plasmid Confers Transferable Carbapenem Resistance in an ST20-K28 *Klebsiella pneumoniae*. Frontiers in Microbiology 13:891807.

17. Gao H, Liu Y, Wang R, Wang Q, Jin L, Wang H. 2020. The transferability and evolution of NDM-1 and KPC-2 co-producing *Klebsiella pneumoniae* from clinical settings. EBioMedicine 51:102599.

18. Wang R, Liu Y, Zhang Q, Jin L, Wang Q, Zhang Y, Wang X, Hu M, Li L, Qi J, Luo Y, Wang H. 2018. The prevalence of colistin resistance in *Escherichia coli* and *Klebsiella pneumoniae* isolated from food animals in China: coexistence of mcr-1 and blaNDM with low fitness cost. Int J Antimicrob Agents 51:739-744.
